# Contraceptive self-injection through routine service delivery: Health worker perspectives from Uganda

**DOI:** 10.3389/fgwh.2022.890017

**Published:** 2022-09-20

**Authors:** Chloe Morozoff, Jane Cover, Allen Namagembe, Damalie Nsangi, Justine Komunyena Tumusiime, Auroras Stout, Jennifer Kidwell Drake

**Affiliations:** ^1^PATH, Seattle, WA, United States; ^2^PATH, Kampala, Uganda

**Keywords:** self-injection, self-care, injectable contraception, DMPA-SC, family planning, Uganda

## Abstract

Self-care reproductive health innovations are increasingly valued as practices that enable women to manage their fertility with greater autonomy. While self-care, by definition, takes place beyond the clinic walls, many self-care practices nonetheless require initial or follow up visits to a health worker. Access to self-care hinges on the extent to which health care workers who serve as gatekeepers find the innovation appropriate and practical. Self-injection of subcutaneous depot medroxyprogesterone acetate (DMPA-SC) is being introduced and scaled in many countries. In late 2018, health workers in Uganda began offering self-injection of DMPA-SC in the public sector, and this study examines health workers' views on the acceptability and feasibility of training women to self-inject. We conducted in-person interviews with 120 health workers active in the self-injection program to better understand provider practices, program satisfaction, and their views on feasibility. A subset of 77 health workers participated in in-depth interviews. Quantitative data was analyzed using Stata (v14) software, and chi square and student t tests used to measure between group differences. Qualitative data was analyzed using Atlas.ti, employing an iterative coding process, to identify key themes that resonated. The majority of health workers were very satisfied with the self-injection program and reported it was moderately easy to integrate self-injection training into routine service delivery. They identified lack of time to train clients in the clinic setting, lack of materials among community health workers, and client fear of self-injection as key challenges. Community health workers were less likely to report time challenges and indicated higher levels of satisfaction and greater ease in offering self-injection services. The relatively high acceptability of the self-injection program among health workers is promising; however, strategies to overcome feasibility challenges, such as workload constraints that limit the ability to offer self-injection training, are needed to expand service delivery to more women interested in this new self-care innovation. As self-injection programs are introduced and scaled across settings, there is a need for evidence regarding how self-care innovations can be designed and implemented in ways that are practical for health workers, while optimizing women's successful adoption and use.

## Introduction

Self-care reproductive health innovations are becoming increasingly recognized and valued as strategies to enable people to manage their fertility with greater autonomy. There is renewed interest in ensuring that appropriately supported self-care interventions are available at scale as part of a fully functional health system. While self-care, by definition, takes place beyond the clinic walls, many self-care practices nonetheless require an initial visit to a health worker (or subsequent follow up with a provider), and are therefore subject to challenges related to how often and to whom the service is made available. One of the key challenges for scale up and access of self-care interventions is therefore understanding the extent to which a new product or practice is acceptable and feasible from the perspective of health care workers, who often serve as gatekeepers (1). While there are myriad health interventions that have demonstrated impact, far fewer have made the transition from research study or pilot program to national scale (1–3).

Self-administration of injectable contraception, made possible by the development of subcutaneous depot medroxyprogesterone acetate (DMPA-SC) in an easy-to-use, all-in-one injection device, is one of two family planning self-care practices (in addition to over-the-counter provision of oral contraceptives) recognized in the WHO guidelines on self-care (4), and which requires initial client self-injection (SI) training. Self-administration of injectable contraceptives can increase women's reproductive autonomy, and reduce potential access barriers such as costly and time-consuming trips to the clinic for reinjection, commodity stock outs, provider absenteeism, and restricted availability of family planning services. Clients who use contraceptives covertly may be better able to maintain privacy through self-injection, and unmarried adolescents may experience less stigmatization by the community or health workers by reducing their health center visits (5, 6). Moreover, the COVID19 pandemic is placing enormous burdens on health systems while increasing the risk of infection for women seeking services at health facilities. In this context, self-care for sexual and reproductive health and beyond is becoming enormously important.

Recent research has shown that self-injection is feasible and acceptable in sub Saharan Africa, and moreover, that self-injection can enable women in diverse contexts to continue uninterrupted injectable use for longer (7–12). While research results were positive, training women to self-inject in the research context can be more resource intensive than is practical in routine service delivery. There is a need to understand how best to structure self-injection programs to function efficiently and effectively outside of a research setting, while maintaining implementation fidelity and quality of care with an already constrained health workforce. As more than 30 countries are currently introducing or scaling self-injection (13), published evidence is critically needed on the best way to implement scalable self-injection programs that are feasible and acceptable for providers and clients (14).

When introducing a new health service innovation, it is important to assess feasibility and acceptability from the providers' perspective to identify challenges and areas for improvement or adaptation before transitioning to scale up. With respect to self-injection and self-care, quality of care is critically important if women are to become autonomous, successful practitioners. We need to learn how to effectively deliver this innovative service, which is unlike anything seen before in the family planning field. Through the Self-injection Best Practices Project evaluation, we explored health worker perspectives and (self-reported) practices in counseling and training women to self-inject. We conducted interviews to understand how providers were implementing the program, their views on the feasibility of training women and adolescents to self-inject, and their overall satisfaction with the program.

## Materials and methods

### DMPA-SC in Uganda

In Uganda, the National Drug Authority approved DMPA-SC for self-injection in 2017 and the country began piloting self-injection through routine service delivery that same year through the Self-injection Best Practices Project. A first round of in-service self-injection training for providers, whose experiences are reported here, began in October 2017 and was completed in December 2017. A total of 230 family planning providers were trained to counsel women for self-injection, including 97 clinic-based providers, 83 community health workers (called Village Health Teams or VHTs) who deliver services in communities, and 50 clinic-based providers who also offer adolescent-responsive services through outreach to safe spaces. Public sector health workers in Gulu, Oyam and Mayuge districts began offering self-injection training for women who express interest at health facilities and in the community through VHTs in early 2018. In a fourth district (Mubende), public sector providers who had previously received training in adolescent-responsive service delivery began offering self-injection training to adolescents through community outreach, in addition to SI services provided at clinics in November 2017.

Under the program's service delivery guidelines, health workers introduce SI in the context of informed choice (one method among many) and include five key topics during training: when and how to reinject, how to securely store devices, how to safely dispose of used units, follow up procedures including resupply and what to do in the event of problems. Women were given two units to take home following the first self-injection, which was supervised by the health worker. Clients were advised to return used units to the health worker at their convenience for safe disposal.

### Study design

A mixed methods approach was used to collect quantitative and qualitative data across the public sector and adolescent channels where self-injection was underway. Health workers active in the self-injection program were recruited to participate in structured surveys about their practices and perceptions of the feasibility and acceptability of the self-injection program. Specifically, structured survey questions were designed to gather information on any outreach or post-training follow up conducted; how training is implemented, including the number of people trained, the type, content, frequency and length of training, and the degree to which training is conducted consistent with service delivery guidelines (including provision of information reinjection timing, storage, disposal, follow up procedures, and side effects counseling); the extent of challenges with service delivery and overall satisfaction with the program. In-depth interviews were used to collect qualitative information from a subset of survey respondents, focusing on provider and (perceived) client receptivity to SI, any restrictions imposed on who is offered SI services, the nature of challenges encountered and suggestions for program improvement.

### Study sites and populations

Sites were selected in a purposive fashion to capture variation in public health system levels (health center level II, III, IV and hospital) and geography. Thirty-four facilities were selected in Mayuge, Gulu, and Oyam districts (10 to 14 in each district), with roughly equal representation by facility level. Sites that had more clients using injectable contraception (within each category of health center) were prioritized.

For the adolescent-responsive program, public sector providers affiliated with Mildmay Uganda, a DREAMS^1^ implementing partner in Mubende district, participated in the pilot introduction. These providers, who had previously received training in adolescent-responsive family planning service delivery, were trained to incorporate self-injection counseling into the contraceptive services they provide to adolescents and young women (AGYW) at “Safe Spaces,” where AGYW could receive HIV prevention and contraceptive services in the community. The same providers who conducted outreach to Safe Spaces also provided SI training to adolescents at their health facilities.

The target population for the evaluation of the public sector program^2^ included both health workers offering services at clinics and VHTs who offered self-injection services in the community. The target providers in the adolescent-responsive program were facility-based health workers offering self-injection services both at public sector facilities and through safe space outreach.

### Sample size

For the structured provider surveys in the public sector, up to five health workers at each of the 34 evaluation sites (20 facilities offering self-injection services on site and 14 facilities affiliated with VHTs offering services in the community) were interviewed, to produce a sample size of 80 providers. For the adolescent-responsive program, we interviewed 40 of the 50 providers who were affiliated with 12 facilities and who received training and provided services to Safe Space adolescent groups in Mubende district. Across all districts, providers were selected in a purposive fashion, based on their level of involvement in the program such that recruitment favored providers who had had greater involvement in self-injection training.

To add more nuanced information about provider practices and opinions, 77 participants among those who participated in the structured surveys participated in in-depth interviews, with 37 of those providers from the public sector channel (20 clinic-based and 17 VHTs) and 40 from the adolescent-responsive program. Whereas, all adolescent-responsive program providers were invited to participate in in-depth interviews, the public sector providers were a convenience sample, selecting those who demonstrated a willingness to provide more detailed input on the program; favorability toward the program did not influence participant selection.

### Recruitment and eligibility

With the permission of the District Health Officer and clinic managers, trained research assistants approached family planning providers at their health facility, or affiliated facility for VHTs, to assess their interest in participating in the evaluation, screen for eligibility and conduct the informed consent process.

Providers were required to be 18 years of age or older, to work in the Mubende, Mayuge, Gulu, or Oyam districts, and to be employed as health workers (i.e., doctor, medical officer, clinical officer, nurse, midwife, nursing assistant, or VHT). Eligibility required that the health worker deliver family planning counseling and services as part of their main responsibilities; that they had been trained in SI counseling; and that they had trained at least 20 women to self-inject since the program launch (approximately 6 months prior). Participants had to be comfortable speaking the language of the interviewer (English, Luganda, Acholi, Langi, or Lusoga).

### Data collection and quality control

Research assistants conducted structured survey interviews^3^ and entered data electronically on mobile phones using Open Data Kit (ODK) software. Data were assessed for quality on a biweekly basis to identify and correct errors. In-depth interviews were subsequently conducted with a subset of providers, audio-recorded and transcribed, with simultaneous translation, if not in English. The first three transcripts from each interviewer were reviewed to assess the quality and completeness of information, and feedback was provided to improve interview quality.

### Data analysis

We analyzed data from the provider surveys using STATA version 14.2. Chi square and student *t*–tests were used to evaluate differences between provider groups, using conventional significance levels of 95% with two-sided tests. We analyzed qualitative data from in-depth interviews using Atlas.ti. The coding process was iterative, with themes developed based on responses rather than a pre-defined coding scheme. Multiple analysts were engaged in the coding process, with disagreements over coding resolved through consensus. We developed memos to summarize key themes, and where appropriate, compared findings by the type of provider (public sector facility-based, VHT, adolescent-responsive provider).

### Ethical considerations

All staff involved in data collection, management, or analysis were trained on research ethics, including confidentiality, informed consent and the voluntary nature of study participation. All health workers who participated in the study provided signed informed consent. The Mulago Hospital Research and Ethics Committee, and the Uganda National Council for Science and Technology (UNCST) approved the study.

## Results

### Background of providers

The description of participating providers is found in Table 1. VHTs who were offering services in the community were more often male than female and significantly older than clinic-based health workers, whether in the public sector or in the adolescent-responsive program. In clinic settings, providers were usually female and nurses or midwives at the level of enrolled nurse (or midwife) or higher. VHTs also played a role in service provision in clinic settings, particularly in the adolescent-responsive program, where they made up nearly one-third of participating providers.

**Table 1:** Background characteristics of health workers (survey data).

	**Public sector Clinic-based HWs *N* = 35**	**Public sector VHTs *N* = 45**	**Adolescent program HWs *N* = 40**
**Age** (median, range)*	32 (24–58 years)	41 (20–62 years)*	33.5 (19–70 years)
**Female gender**	82.9% (29)	42.2% (19)*	92.5% (37)
**Job title**			
VHT	20.0% (7)	100% (45)	32.5% (13)
Nursing assistant	11.4% (4)	-	2.5% (1)
Enrolled Midwife/Nurse	34.3% (12)	-	57.5% (23)
Registered Midwife/Nurse	25.7% (9)	-	5.0% (2)
Comprehensive EM/EN or	8.6% (3)	-	-
Double trained RM/RN Peer mother	- -	- -	−2.5% (1)
**Level of facility**			-
HC I (VHT)	-	100% (45)	
HC II	51.4% (18)	-	25.0% (10)
HC III	37.1% (13)	-	75% (30)
HC IV or hospital	11.4% (4)	-	-
**Number years in family planning** (median, range)	5 (1–26 years)	5 (0.6–12 years)	7 (0.5–35 years)

*Indicates significant difference at the P < 0.05 level from public sector clinic-based health workers and adolescent-responsive program health workers.

Health workers (HWs), Village Health Teams (VHT), Health Center (HC), Enrolled Midwife (EM), Enrolled Nurse (EN), Registered Midwife (RM), Registered Nurse (RN).

### Access to self-injection training

When asked *via* in-depth interviews to whom they offer self-injection training, most of the 77 providers reported offering self-injection to all types of clients, with some spontaneously opining that informed choice dictates that any client who is interested can receive self-injection training.

That said, with probing, some providers admitted to restricting access for younger women. Eleven providers, eight of whom were VHTs, reported they impose age restrictions on unmarried adolescents, particularly those who are still in school and have not yet given birth, and will, in lieu of training them, counsel them to abstain or use condoms. In all cases, their concerns focused on adolescent sexuality and misperceptions about whether young women can safely use injectable contraception, rather than apprehension over whether adolescents could manage self-injection.

“*We have adolescents who come and consult you and tell you they'd like an injection. I ask them how old they are. If she says seventeen years I say eeeeeeh. I educate and counsel her that she should be patient because you should not engage in sexual acts; at least use condoms instead of this injection. Then she shares that the partner refused condoms. I counsel her that she should tell her partner not to use an injection at an early age. A person to start off the injection at the earliest it should be eighteen to nineteen years, though at times I get clients as young as sixteen years. To such young ages I counsel first and educate. I cannot stop you because you have started sleeping around, so I advise them to use this other method of condoms.” VHT, HC II, Adolescent-responsive program*

A smaller number (seven) indicated that they do not offer self-injection training to women who are using discreetly, largely out of concerns that injections at home may be discovered.

“*There are sometimes when a woman escapes to my home to get the injection at night because they fear their husbands, so I just inject but not train them.” VHT, public sector (community based)*

### Provider training practices

Table 2 details the frequency and style of trainings reported by health workers. Most health workers in the public sector and the adolescent-responsive program had trained clients to self-inject in the month prior to their survey interview; only seven providers (out of 120 surveyed) reported that they had not trained anyone in the recent time frame. When asked why they had not trained any clients, five reported that they were away from their site in the past month, one did not have time, and one was not interested in training clients (data not shown). Clinic-based providers and community-based VHTs in the public sector trained a similar number of clients in the previous month (median of 6 and 10 respectively); adolescent-responsive program providers trained a significantly higher number (median of 25).

**Table 2:** Provider-reported frequency and style of self-injection training (survey).

	**Public sector clinic-based HWs *N* = 35**	**Public sector VHTspar *N* = 45**	**Adolescent program HWs^+^ *N* = 40**
**Trained clients in the past month**	82.9% (29)*	100% (45)	97.5% (39)
**Median number of clients trained past month:** (range)	6 (0–44)	10 (2–38)	All clients: 25 (0–100)^@^ Adolescents: 10 (0−60)
**Training format usually used**			
Group training only	17.1% (6)	20.0% (9)	28.2% (11)
Individual training only	11.4% (4)	11.1% (5)	2.6% (1)
Both types	71.4% (25)	68.9% (31)	69.2% (27)
**Health workers who usually conduct individual trainings**			
	*n* = 29	*n* = 36	*n* = 28
Median number of trainings/week (range)	3 (0-−15)	4 (1-−13)	4 (1-−35)
Median training duration in minutes (range)	25 (10-−45)	22.5 (5-−90)	20 (10-−50)
**Health workers who usually conduct group trainings**			
	*n* = 31	*n* = 40	*n* = 38
Median number of trainings/week (range)	2 (0-−5)	2 (0-−4)	1.3 (0-−10)
Median group size (range)	5.0 (3-−40)	6.5 (3-−30)	20 (5-−150) ^@^
Median training duration in minutes (range)	30 (15-−120)	45 (15-−150)	35 (5-−120)

^**+**^Adolescent-responsive program providers were specifically asked about their experience training adolescents.

*Significant differences from VHTs and adolescent-responsive program providers at the P < 0.05 level.

@Significant difference from public sector clinic-based providers and VHTs at the P < 0.05 level.

Approximately 70 percent of providers conducted training in both group and one-on-one sessions, with no difference by service delivery approach. While the program guidelines left the determination of training format up to providers, they were advised to limit the group size to 10 or fewer. Providers reported conducting approximately 3–4 individual trainings per week, with a median duration of 20 min, and 1–2 group trainings, with a median duration of 40 min. The duration of group training sessions varied somewhat (though not significantly) by service delivery approach, from 30 min at public clinics to 35 min at adolescent safe spaces to 45 min in community settings led by VHTs. The range of duration for these group sessions was large, from 5 to 150 min. The median size of a training group at an adolescent safe space was 20 clients, significantly higher than in the public sector channel, where the average was five or six clients.

In the public sector, providers were either offering self-injection services in the clinic, or if VHTs, in the community. In the adolescent-responsive program, providers were supported to offer services both in the clinic and at adolescent safe spaces in the community. When providers in the adolescent-responsive program were asked where and how they usually train adolescent clients, 46% reported they train adolescents at safe spaces only, 13% train adolescents at clinics only, and 41% reported that they train at both locations. In addition, a slight majority (55%) reported that they only train adolescents with peers, as opposed to training adolescents in groups with women of all ages (45%, data not shown).

**Figure 1:**
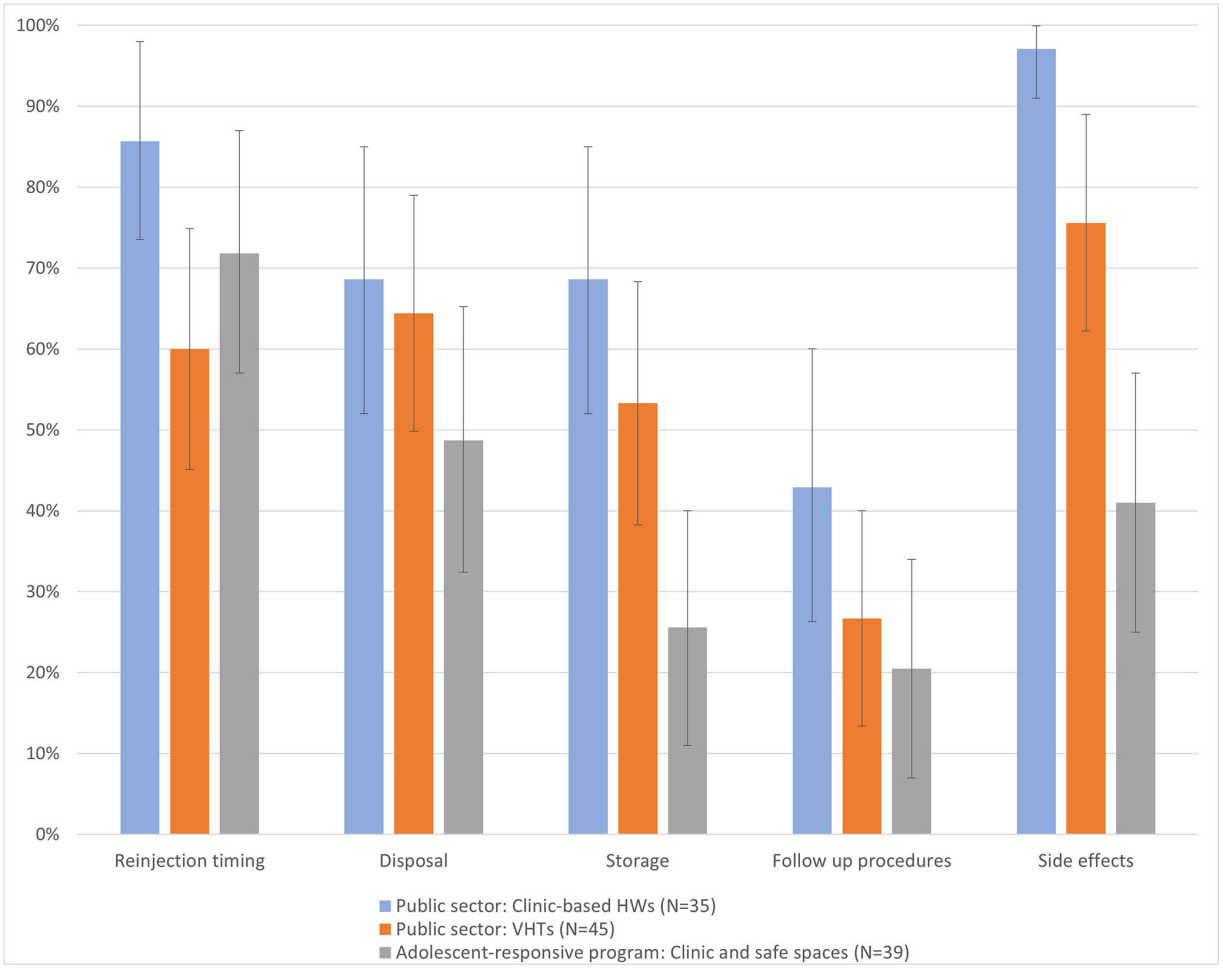
Self-injection topics discussed by health workers during client training.

While the format of training (group or individual) was left to providers to decide, service delivery guidelines identified key additional subjects, beyond injection technique, that should be included for a quality self-injection training, including storage, disposal, reinjection timing, side effects and follow up. The evaluation assessed the extent to which providers reported implementing training consistent with the guidelines by asking them to describe what information they cover during training. We have reported additional topics of interest in Figure 1. Across all topics, public sector facility-based providers were the most likely to report covering key training topics, though the between group differences were often not significant. The differences were largest for storage and side effects content; side effects were nearly always discussed by clinic-based providers (97%), usually discussed by VHTs (76%) but discussed less than half of the time among providers in the adolescent-responsive program (41%).

### Helping clients overcome challenges to self-injecting

Client fear of self-injection was a challenge raised by all provider groups in the structured surveys as well as in more detail in the in-depth interviews. When providers were asked what women struggle with the most when learning to self-inject, the most common response across providers was that women fear injecting themselves, followed by challenges activating the DMPA-SC device (Table 3). Providers in the adolescent-responsive program were specifically asked to report on what their adolescent clients are most challenged by, whereas public sector providers were asked about clients without regard to age. A significantly higher percentage of adolescent-responsive program providers (82%), reported that adolescent clients struggled with fear, compared to public sector clinic (54%) and community-based providers (60%).

**Table 3:** Health worker-reported challenges for clients learning to self-inject (survey).

	**Public sector clinic-based HWs *N* = 35**	**Public sector VHTs *N* = 45**	**Adolescent program HWs *N* = 39**
Fear injecting herself	54.3% (19)	60.0% (27)	82.1% (32)*
Activating the device	51.4% (18)	51.1% (23)	48.7% (19)
Holding the device by the port	28.6% (10)	33.3% (15)	15.4% (6)
Squeezing slowly	17.1% (6)	28.9% (13)	18.0% (7)
Shaking the device adequately	11.4% (4)	15.6% (7)	5.1% (2)
Reading the calendar	5.7% (2)	6.7% (3)	(0)
Locating an injection site	2.9% (1)	4.4% (2)	2.6% (1)
Tenting the skin	2.9% (1)	4.4% (2)	12.8% (5)
Privacy at home	0.0% (0)	2.2% (1)	10.3% (4)
Side effects	0.0% (0)	0.0% (0)	7.5% (3)

*Significant difference from public sector providers and VHTs at the P < 0.05 level.

In the in-depth interviews, when asked to elaborate about fear as a challenge, some health workers indicated that fear is only an initial challenge that clients overcome with counseling or after hearing about self-injection from their peers. Other health workers noted that fear of self-injection is a persistent barrier for some clients, with indication from a few providers that those who are older and who have not attended school tend to be more fearful.

“*At first, I was questioning myself how someone can give herself an injection because I know women are so fearful compared to men, so I thought they won't make it at the start of the program, but afterwards the fear has subsided. The women who are already self-injecting are really helping me a lot to encourage their fellow women in the community.”*-*VHT, public sector (community based)*

“*The demand for self-injection is good, only that there are those who can't [self-inject]. Especially if you find someone who didn't go to school, she becomes so fearful to self-inject.” -VHT, public sector (community based)*

The most common reasons offered in the in-depth interviews for denying the opportunity to self-inject independently were failure to demonstrate injection competence (*n* = 26), and excessive fear (*n* = 16), though these responses were mentioned by less than half of providers.

“*If she fails to grasp the steps we go through while self-injecting well, then I don't give her an opportunity [to self-inject at home]. There are those who fear a lot: she tries to hold it but she herself fears, so she also doesn't get to self-inject.” –Midwife, HC III, Adolescent-responsive program*

“*I first test her. I do not just give the service after the training. We use the training model and device while following the steps on the job aid. I hold the job aid chart at my chest after handing her the practice materials for her to show me and follow me. Adolescents I see maybe because they are still in school, they grasp quickly and have strong hearts but these (older) women are so fearful. Adolescents do it confidently and speed up.” -VHT, HC II, Adolescent-responsive program*

### Perceived benefits of self-injection

Providers were readily able to identify benefits to offering self-injection services, and chief among them, cited by about three fourths of providers who participated in in-depth interviews, was the potential to reduce the workload at the clinic.

“*I feel it (self-injection) is a good thing because it reduces my work load. Because sometimes I really have a lot of clients to work on, and if this mother is able to help herself, it reduces what I have to do. I need to supervise her that first time and then the next time I know she is not really going to be there except if she is having a proble*m.” -*Enrolled midwife, HC III, Public sector*

In terms of the benefits to the client, just under half of providers noted that clients save time and money by not returning as frequently to the clinic, and about one in five noted that self-injection can enhance privacy, which is critical for women who are using family planning discreetly. In particular, adolescent-responsive program providers mentioned enhanced confidentiality as a benefit to young, unmarried women.

“*It (self-injection) has been helpful for privacy as at times they (adolescents) come to the facility. You see so-and-so's daughter, but once you train her and she goes home, she can be able to inject herself.” -Enrolled midwife, HC III, Adolescent-responsive program*

“*… others fear their husbands, but when she comes and we give her Sayana*^4^*, when she has learnt to self- inject then she goes and self-injects at her home. So, when three months have passed, she waits for her husband, the one she fears, to go away then she injects herself, which helps her not get unwanted pregnancy.”*-*Nursing assistant, HC II, Public sector*

### Challenges with integration into routine services

Providers were asked to describe their experience with the self-injection program, evaluate the ease with which they could integrate self-injection training into their work and rank their satisfaction with the program. Providers reported moderate ease integrating self-injection into the services they offered (Table 4). VHTs reported the greatest ease, with 49% reporting it was “very easy” to add self-injection, as compared with about one-third of clinic-based providers in the public sector (34%). Providers in the adolescent-responsive program reported somewhat greater ease adding self-injection to safe space outreaches (36%), compared to clinic-based services (28%). At times, a lack of materials (such as job aids) inhibited the availability of self-injection training; lack of materials was somewhat common among public sector clinic providers (17%), but more commonly mentioned by VHTs (29%). A majority of providers in the public sector and adolescent-responsive program reported challenges offering self-injection training due to workload. In fact, 35 to 60 percent of providers stated that they sometimes, often, or almost always are unable to offer self-injection training because they are too busy with other duties.

**Table 4:** Health worker perceptions of the self-injection program (survey).

	**Public sector clinic-based HWs *N* = 35**	**Public sector VHTs *N* = 45**	**Adolescent program** **HWs** ***N*** = **40**	
Ease of adding self-injection			At clinics	Safe spaces
Very easy	34.3% (12)	48.9% (22)	27.5% (11)	36.1% (13)
Somewhat easy	45.7%(16)	37.8% (17)	40.0% (16)	50.0% (18)
Somewhat difficult	20.0% (7)	13.3%(6)	27.5% (11)	13.9% (5)
Very difficult	0	0	5.0% (2)	0
No training offered due to lack of personnel/too busy (sometimes or often)	60.0% (21)*	35.6% (16)	57.5% (23)	
No training offered due to lack of materials (sometimes or often)	17.1% (6)	28.9% (13)	2.5% (1)^@^	
No safe space outreach offered due to lack of transport (sometimes or often)	NA	NA	62.5% (25)	
Satisfaction with program			
Very satisfied	62.9% (22)	77.8% (35)	57.5% (23)	
Somewhat satisfied	25.7% (9)	17.8% (8)	30.0% (12)	
Somewhat unsatisfied	11.4%(4)	4.4% (2)	12.5% (5)	
Very unsatisfied	0	0	0	

*Difference between public sector providers and community based VHTs was significant (p < 0.05).

^@^Differences between providers in the adolescent-responsive program and public sector (both clinic and community based) was significant (p < 0.05).

Through in-depth interviews, providers elaborated on specific challenges, explaining that there are often periods of high client load in the clinic, and it is during these busy periods that it is challenging to offer self-injection. Other comments suggest that lack of time for training may compromise quality.

“*It has been somewhat difficult in such a way that self-injection clients need more time. First, they need time for counseling and secondly, they need time for the training, to observe them offering the method. Sometimes there is also other work in the facility—there may be the deliveries and the emergencies. You tell the family planning clients to wait a bit, while you are taking an emergency, but you come and find they have disappeared.” –Nurse, HC IV, public sector*

“*At times it could be really a lot [to offer self-injection training], especially when training one-on-one with a client and yet you really have a queue of clients. It has been hard to balance my usual workload at the clinic. Not every day is a busy day but those other busy days, it can be a little bit of a challenge.” -Nurse-midwife, HC IV, public sector*

“*It takes a lot of time to train women, so we just inject sometimes without training.” - Midwife, HC III, adolescent-responsive program*

Despite the challenges, the majority of health workers reported that they were ‘very satisfied' with the self-injection programs: 63% in the clinic-based provider group, 78% in the VHT worker group, and 58% among the providers in the adolescent-responsive program (Table 4).

### Provider recommendations for self-injection programs

Not all providers interviewed (*n* = 77) provided recommendations for the program, but those who did generally centered on ways to improve training and community sensitization. Regarding training, some providers suggested the need to train more staff (*n* = 13), a few emphasizing the need to train more VHTs; some recommended increasing the duration of training for providers (*n* = 11); and some suggested offering refresher training and/or supportive supervision (*n* = 15).

“*As staff we should get a refresher course and they should also train new staff, because some of the old staff have transferred facilities.” -Midwife, HC III, Adolescent-responsive program*

“*I think it would be helpful if, once in a while, my supervisor came back to check on me. Because sometimes after a training, after I have been doing it on my own, there may be parts I have not been doing well. I think if am supervised again I can make it better.” -Midwife, HC III, Public Sector*

Regarding community awareness or engagement around self-injection, a few providers recommend conducting more sensitization activities in the community either in the form of community dialogues and/or radio (*n* = 8), including greater focus on men. They noted that most clients had not heard of self-injection prior to arriving at the clinic, VHT or safe space. Specific suggestions were to provide branded materials so the community knew they were offering self-injection, and having more VHTs or safe space mentors provide self-injection training.

“*This program can be improved by intensifying community dialogues or intensifying outreaches for service provision, because usually it is challenging for the community—the women deep in the village—to come to the health center. It would also be helpful to intensify the radio talk shows and provide family planning at outreach points where there is HIV testing.” -Nurse, HC IV, Public Sector*

## Discussion

This study is one of the first to share provider experiences with routine implementation of self-injection outside of a research setting. Our findings are generally encouraging with relatively high levels of reported satisfaction with the program, particularly among community health workers. In this sense, our findings complement those from a small qualitative study of Malawian providers, who also viewed self-injection (implemented through an RCT) in a positive light (15).

This study also reveals a number of program implementation adaptations, highlighting the importance of program monitoring and supervision to maintain intervention fidelity and service quality when introducing a new practice. Specifically, we found training group sizes that exceeded the service delivery guidelines of < 10 (in a few cases with groups larger than 100), particularly in the adolescent-responsive program when self-injection training was offered at Safe Spaces. Based on observations by the research team, these larger group trainings (which were predominately in the adolescent-responsive channel) were likely to have been health talks, rather than specific and detailed trainings. The challenge of offering self-injection training to large groups in the adolescent responsive program (at Safe Spaces) may also explain differences in training quality, and specifically, the omission of information on side effects (as described below). Although these Safe Space outreach events may function well to raise awareness of self-injection among an underserved population, confidential service delivery, which is a central tenet of a rights-based quality of care framework in family planning (16), requires a separate, private space that allows adolescents (or any clients) to speak with providers confidentially and to receive one-on-one supervision and guidance while self-injecting for the first time.

While individualized training in self-injection may be appealing from a quality-of-care standpoint, the duration of comprehensive training, and the finding that a majority of clinic-based providers are sometimes or often unable to offer training due to workload constraints, requires a more efficient approach. If self-injection is to be made available to all women who would like to adopt it, group training, with provision for a shorter one-on-one consultation, is a reasonable program adaptation to reduce time burdens on providers. Since VHTs were less likely to report time challenges and indicated higher levels of satisfaction and greater ease in offering self-injection services, involving more community and clinic-based VHTs is likely to increase program reach. Increasing self-injection outreach and training through community health workers and Safe Space mentors was specifically recommended by providers in the program. Note that, for one of the early (and overall very positive) studies of self-injection in Malawi, the majority of providers were community health workers (12). When expanding to involve more VHTs, attention will be needed to the supply chain, including a consistent supply of job aids, given that lack of self-injection materials was identified as a relatively common challenge by VHTs in this program.

In addition to limiting the availability of self-injection training, workload constraints may also impact training quality. Some in-depth interview comments imply that an abbreviated training approach was a response to time constraints and a heavy workload. Specifically, we saw differences in the comprehensiveness of training, and particularly notable was the omission of discussion of side effects in the adolescent-responsive program. Method side effects are a common cause of discontinuation (17), and side effects counseling has been shown to improve continuation (18). High rates of method discontinuation are common among adolescents, which may be tied, at least in part, to inadequate side effects counseling (19). A training video for clients, tested in the private and public sector in a separate study (results forthcoming), shows promise as a complement to provider training, and may serve to standardize training, ensuring that all topics are covered, while reducing the time burden on providers.

Ensuring training comprehensiveness and enhancing overall training quality will likely increase client self-confidence and reduce fear, identified across provider groups as the greatest difficulty that clients face. Its notable that our companion study of client experiences in the SI program reinforces providers' impression that quite a few clients are fearful of self-injecting (20). In our focused study on the adolescent experience, adolescents were less confident in their ability to self-inject, less likely to report that giving the injection was “very easy,” and felt less prepared to self-inject independently (21). Helping clients to overcome injection anxiety may be challenging to navigate, however, as providers must find a balance: encouraging a nervous client through age appropriate counseling, without pressuring her into an option that might not be right for her.

We found that a small number of providers expressed reluctance to train adolescent women, highlighting persistent challenges with provider bias against adolescent contraceptive use, and hormonal contraceptive use specifically. These findings are consistent with a previous study in Uganda, which showed reluctance to offer adolescent women contraception (due to adolescent sexuality concerns), injectables (related to infertility impacts) as well as self-injection (perceptions of lack of maturity) (22). Monitoring to track adolescent access to self-injection (mode of injectable administration by age) is necessary if we are to evaluate scale up in terms of equitable access for young women. Because training alone has not been shown to reduce provider bias, refresher training and supportive supervision (both programmatic recommendations offered by providers themselves) should be accompanied by specific provider bias reduction initiatives such as values clarification exercises, mentoring and dissemination of guidelines for serving youth (23). These health system-strengthening interventions will be important going forward not only to improve equitable access for adolescents but to ensure that self-injection program adaptations to the local context maximize quality service delivery and enable women to become autonomous, successful self-injectors.

Finally, we note that for many of the issues described above, we see differences, often statistically significant, between types of providers with respect to experiences with, and perceptions of, the SI program. To sum up, VHTs expressed higher satisfaction and ease of incorporating SI services into their family planning offerings than did the clinic-based providers. VHTs were however, more likely to express challenges with a lack of materials and were less likely to discuss side effects. We also saw more VHTs express reluctance to serve adolescent clients, suggesting the possibility of provider bias. While the data is qualitative and therefore merely suggestive, it is consistent with a finding from our companion paper focusing on the adolescent experience which showed significantly fewer adolescents receiving services from VHTs (21). We note that the VHTs in this study were older on average and significantly more likely to be male than were clinic-based providers. It is difficult to know the extent to which adolescents may avoid VHTs due to gender, age, confidentiality considerations, and/or fear of judgment, as opposed to VHTs actively turning away adolescents seeking self-injection (and/or contraception more generally); it is likely that both factors are at play. In any event, our results reinforce the importance of building the capacity of community health workers to provide high quality family planning services and to improve their receptivity to serving adolescents in an informed and judgement-free fashion; more generally, our results highlight the important role that community health workers can play in self-injection service delivery.

## Limitations

To understand how the program was being implemented, the sampling strategy for the evaluation prioritized providers who had trained at least 20 clients over the first 6 months of the program. While this criterion maximized information about program implementation, the strategy over-sampled providers who were committed and actively involved. The reported satisfaction and ease of integration of self-injection into routine services is likely inflated due to our sampling approach. Second, we employed purposive sampling for the in-depth interviews, selecting those willing to discuss experiences in detail to collect more vibrant and expansive data, and avoiding respondents who would provide terse responses because they are rushed, bored, or indifferent. There are trade-offs with this strategy however, and it is possible that our sample includes a preponderance of providers with more extreme views. Third, as this evaluation was based on a small-scale introduction of self-injection, the sample size is small, increasing the risk of type 2 error (in this case, failure to detect significant differences between provider groups). Finally, the findings are self-reported, and therefore subject to courtesy and social desirability bias.

## Conclusion

As self-injection programs are introduced and scaled across settings, there is a need to collect and share evidence and guidance regarding how self-injection programs and supported self-care innovations can be designed and implemented in ways that optimize women's successful adoption and use. Our findings suggest that, from the provider perspective, it is both feasible and acceptable to offer self-injection services through multiple channels and by different cadres of providers within the public sector. This paper complements two companion pieces that focus on the experiences of self-injecting women (20) and adolescents (21) under the Self-injection Best Practices Project. Collectively, this mixed methods evaluation is generating evidence and guidance that decision-makers and managers in Uganda and globally can use to design and implement effective and practical self-injection programs. Program planners should consider these findings alongside client experiences to tailor programs to be acceptable, feasible, and of high quality.

## Data availability statement

The raw data supporting the conclusions of this article will be made available by the authors, without undue reservation.

## Ethics statement

The studies involving human participants were reviewed and approved by the Mulago Hospital Research and Ethics Committee; Uganda National Council for Science and Technology (UNCST). The participants provided their written informed consent to participate in this study.

## Author contributions

JC, AN, CM, DN, and JKT were involved in the study design, including development of the protocol and design of data collection instruments. AN, DN, and JKT supervised data collection. CM and JC led the quantitative data analysis and AS engaged in the qualitative data analysis. JC, AN, CM, DN, JKT, AS, and JKD were involved in the interpretation of results, writing and revision of the article. All authors have approved this manuscript.

## Funding

This work was supported by the Bill and Melinda Gates Foundation, Seattle, WA under Grant Number: 1154309. The funding source did not play a role in study design, the collection, analysis, and interpretation of data, the writing of the report, and the decision to submit the article for publication. The Bill and Melinda Gates Foundation provide funds for open access publication.

## Conflict of interest

All authors were employed by PATH. The authors declare that the research was conducted in the absence of any commercial or financial relationships that could be construed as a potential conflict of interest.

## Publisher's note

All claims expressed in this article are solely those of the authors and do not necessarily represent those of their affiliated organizations, or those of the publisher, the editors and the reviewers. Any product that may be evaluated in this article, or claim that may be made by its manufacturer, is not guaranteed or endorsed by the publisher.

## Footnotes

^1^The DREAMS (Determined, Resilient, Empowered, AIDS-free, Mentored and Safe) partnership is a public-private partnership to reduce HIV infection among adolescent girls and young women (AGYW) in the highest HIV burden countries. “Safe Spaces” are community locations specifically designated as sites for HIV and FP service delivery targeting adolescents and young women.

^2^For simplicity in this article, we distinguish the public sector program from the adolescent-responsive program, to recognize the difference in self-injection service delivery approaches. However, all providers involved in the pilot were employed in the public sector.

^3^The instrument employed was comparable, whether for public sector or Adolescent-responsive program, but with the addition of some adolescent-specific questions (i e., number of adolescents trained, etc.).

^4^Sayana^®^ Press is the brand name of the DMPA-SC product used in Uganda, manufactured by Pfizer. In some contexts, providers and clients refer to the product by its brand name, or Sayana^®^ for short.
